# Miliaire cristalline généralisée

**DOI:** 10.11604/pamj.2016.25.163.9745

**Published:** 2016-11-16

**Authors:** Sara Elloudi, Fatima Zahra Mernissi

**Affiliations:** 1 Hôpital Universitaire Hassan II, Service de Dermatologie et Vénérologie, Fès, Maroc

**Keywords:** Miliaire sudorale, miliaire cristalline, état fébrile, Miliara sweat, crystalline miliara, fever

## Image en médecine

La miliaire sudorale est une manifestation cutanée bénigne liée à une rétention sudorale secondaire à une obstruction des canaux sudoraux. Selon le niveau d'obstruction, on distingue la miliaire cristalline, la miliaire rouge et la miliaire profonde. Nous rapportons un patient de 59 ans, sans antécédents pathologiques notables, hospitalisé au service de réanimation pour une confusion fébrile en rapport avec une méningoencéphalite herpétique. Le patient a présenté des lésions vésiculeuses diffuses au niveau de tout le corps, de contenu clair, fermes à la palpation, reposant sur une peau saine, correspondant à une miliaire cristalline. La miliaire cristalline survient lors des affections fébriles aiguës avec une hypersudation brutale. Elle se manifeste par de multiples lésions vésiculeuses superficielles asymptomatiques à contenu clair, comme des gouttes de rosée reposant sur une peau saine, siégeant sur le tronc et l'abdomen. La forme généralisée sur tout le corps, comme le cas de notre patient, est rare. L'obstruction se fait au niveau de la couche cornée. La miliaire cristalline guérit spontanément en quelques heures en laissant une desquamation furfuracée, comme c'était le cas chez notre patient après régression du syndrome fébrile.

**Figure 1:**
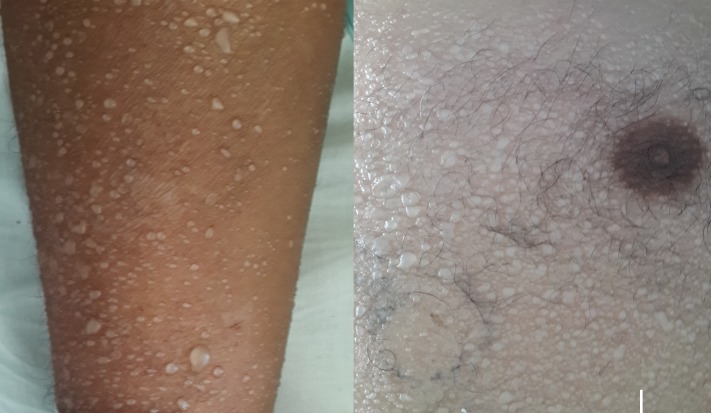
Vésicules claires fermes reposant sur une peau saine, disséminées à tout le corps

